# Application of Tubular Reactor Technologies for the Acceleration of Biodiesel Production

**DOI:** 10.3390/bioengineering9080347

**Published:** 2022-07-27

**Authors:** Omojola Awogbemi, Daramy Vandi Von Kallon

**Affiliations:** Department of Mechanical and Industrial Engineering Technology, University of Johannesburg, P.O. Box 524, Johannesburg 2006, South Africa; dkallon@uj.ac.za

**Keywords:** tubular reactor, biodiesel, catalyst, transesterification, feedstock, reactor technologies

## Abstract

The need to arrest the continued environmental contamination and degradation associated with the consumption of fossil-based fuels has continued to serve as an impetus for the increased utilization of renewable fuels. The demand for biodiesel has continued to escalate in the past few decades due to urbanization, industrialization, and stringent government policies in favor of renewable fuels for diverse applications. One of the strategies for ensuring the intensification, commercialization, and increased utilization of biodiesel is the adaptation of reactor technologies, especially tubular reactors. The current study reviewed the deployment of different types and configurations of tubular reactors for the acceleration of biodiesel production. The feedstocks, catalysts, conversion techniques, and modes of biodiesel conversion by reactor technologies are highlighted. The peculiarities, applications, merits, drawbacks, and instances of biodiesel synthesis through a packed bed, fluidized bed, trickle bed, oscillatory flow, and micro-channel tubular reactor technologies are discussed to facilitate a better comprehension of the mechanisms behind the technology. Indeed, the deployment of the transesterification technique in tubular reactor technologies will ensure the ecofriendly, low-cost, and large-scale production of biodiesel, a high product yield, and will generate high-quality biodiesel. The outcome of this study will enrich scholarship and stimulate a renewed interest in the application of tubular reactors for large-scale biodiesel production among biodiesel refiners and other stakeholders. Going forward, the use of innovative technologies such as robotics, machine learning, smart metering, artificial intelligent, and other modeling tools should be deployed to monitor reactor technologies for biodiesel production.

## 1. Introduction

**Figure 1:**
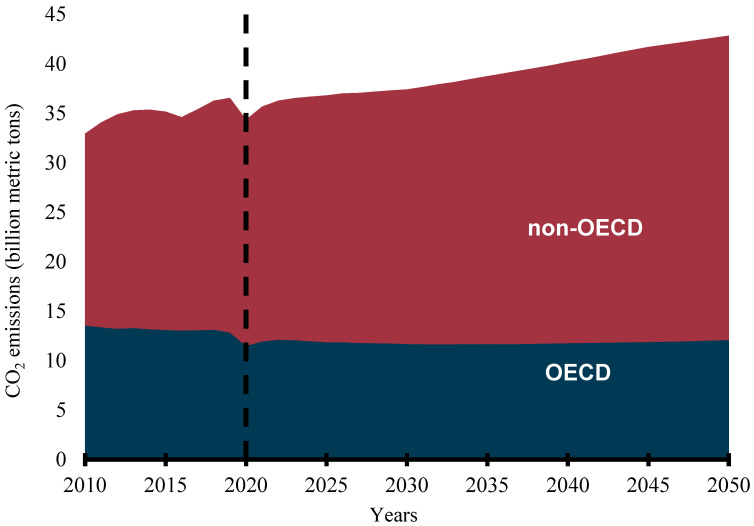
Energy-related CO_2_ emissions (billion metric tons).

Rapid population growth, industrialization, urbanization, economic growth, social development, and technological advancement have continued to increase energy consumption worldwide. Credible available statistics show that the global primary energy consumption was 109,583 terawatt hours (TWh) in 2000, became 140,562 TWh in 2010, and rose to 162,194 TWh in 2019 [1]. The International Association for Energy Economics has predicted that primary energy consumption will grow by over 39% over the next 20 years [2] while the International Energy Agency predicts more than a 50% increment in global primary energy consumption by 2050 [3]. The continuous energy demand is premeditated on the importance of the availability of a sustainable, reliable, affordable, and accessible energy supply to the social, economic, and industrial development of any country. However, most of the world’s energy is sourced from fossil fuels. REN21, an international renewable energy policy network, reports that about 80% of the global energy mix still comes from fossil fuels [4]. Consumption of fossil-based (FB) fuels in the transportation, industrial, agricultural, commercial, household, and power generation sectors has continued to increase despite the efforts to stem the tide by various governments, organizations, and interests. The emission of carbon dioxide (CO_2_) from energy-related applications is predicted to continue to increase globally. Though the CO_2_ emissions from the Organization for Economic Cooperation and Development (OECD) countries have remained flat, that of the non-OECD nations are predicted to continue to rise, as shown in Figure 1 [5]. This trend is not likely to change in the foreseeable future as the share of FB fuels in the transportation sector has been predicted to be no less than 88% in 2040 [5]. This has led to the release of toxic emissions and other serious environmental degradation concerns and exacerbated global warming and climate change.

To stem this tide, researchers have shown more interest and committed more resources in terms of investments to finding sustainable and environmentally friendly alternatives to the dangerous FB fuels. Primarily, attention has been placed on finding renewable energy sources such as solar, hydroelectric, hydrogen, wind, biodiesel, green diesel, bioethanol, biofuels, and biomass [6]. Biodiesel is seen as one of the sustainable and affordable replacements for FB fuels for various applications. Biodiesel, also known as fatty acid methyl ester, is a renewable, cost-effective, and biodegradable liquid fuel synthesized from vegetable oils, recovered restaurant oil, animal fat, tallow, non-edible plant oil, waste cooking oil, microalgal plants, and other triglycerides-bearing feedstocks [7]. The use of biodiesel is to remedy the uninspiring performance of FB fuels in internal combustion engines, emission of toxic gases, and the impacts on the environment and humanity in general. It is believed that the application of biodiesel, particularly in internal combustion engines will improve engine performance, reduce tailpipe emissions, and mitigate the unpleasant effects on the environment and global health [8]. Though there are a few drawbacks related to the application of biodiesel, the fact that the benefits of biodiesel far outweigh these shortcomings makes biodiesel a sustainable alternative to FB diesel fuel (Table 1).

**Figure 2:**
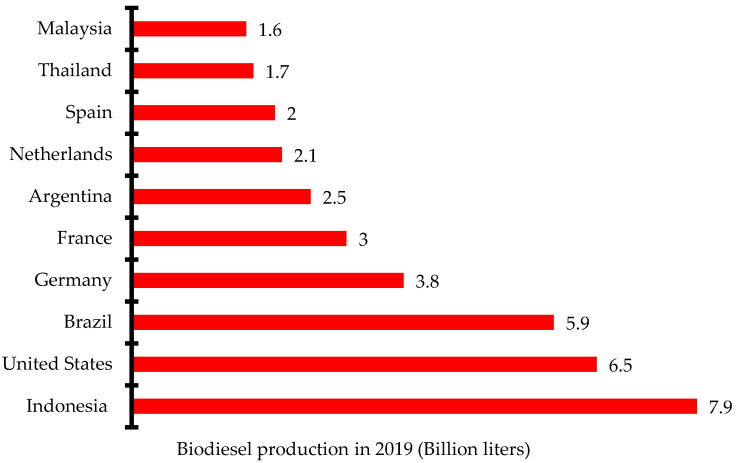
Ten leading biodiesel producers in 2019.

**Figure 3:**
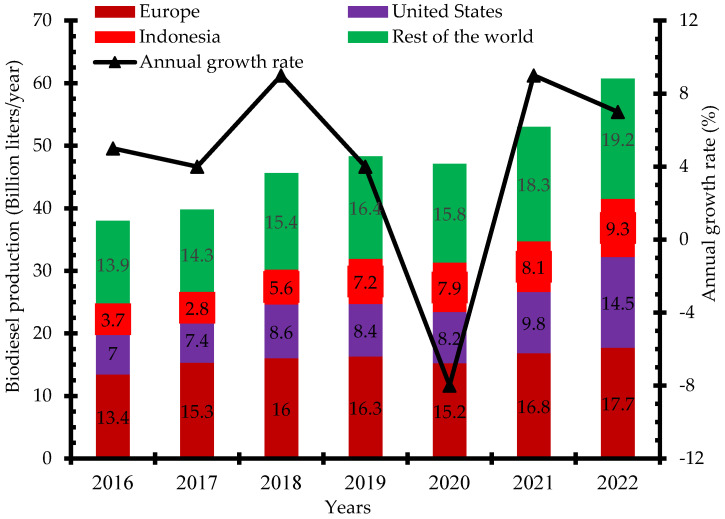
Global biodiesel production 2016–2022 (billion liters/year).

There has been increased research and investment in the production of biodiesel over the past few years to be able to meet up the global demand. Indonesia, the United States, and Brazil are the three leading biodiesel producers in 2019 with 7.9 billion liters, 6.5 billion liters, and 5.9 billion liters, respectively (Figure 2) [12]. Global biodiesel production increased from 38 billion liters in 2018 to 48.3 billion liters in 2019 and 60.7 billion liters in 2022. The annual growth rate of biodiesel production increased from 4% in 2017 to 9% in 2021. The negative growth rate recorded in 2020 was due to the impact of the dreaded COVID-19 pandemic that restricted movement and slowed down economic activities in most countries (Figure 3) [13]. Concerted efforts including research and development, infrastructure, policy framework, incentives, and investments have been put in place to ensure the increased production of biodiesel for diverse applications.

### Motivation, Aim, and Objectives

Investigations have been conducted on the various technologies for the synthesis of biodiesel in commercial quantities. Chuah et al. [14], Kant Bhatia et al. [15], and Bashir et al. [16] studied the various advanced technologies for low-cost and high conversion yield for biodiesel production. They reported that the intensification of production techniques such as microwave, ultrasonic radiation, cavitation, plasma discharge, transesterification, pyrolysis, supercritical, and emulsification was ecofriendly and resulted in a higher product yield and lower wastes during biodiesel production. Roick et al. [17] investigated the various thermochemical, biomechanical, chemical, and other novel technologies for the commercial production of biodiesel and reported that these technologies were viable from the economic and ecological points of view. The works of Mohiddin et al. [18], Okolie et al. [19], and Lv et al. [20] investigated the impact of feedstocks, catalysts, production methods, and production infrastructure on the commercialization of biodiesel. They reported that to achieve the return on investment in biodiesel production, the choice of feedstock, catalysts, and selection of converting infrastructure plays an important role. The choice of reactor technology and other processing parameters affects the conversion rate, cost of production, and yield of biodiesel irrespective of the types of feedstock and catalyst [21].

Despite these reported cases, there are ongoing studies on how to meet the continuous demand for biodiesel for diverse applications. There is near unanimity of opinions on the need to interrogate and carry out further investigations on the mechanisms for achieving the acceleration of biodiesel production through the deployment of reactor technologies. In addition, there is an urgent need to extend the frontiers of available knowledge on the infrastructure for accelerated biodiesel generation to meet the ever-growing demand. These form the motivations for the current study. The aim of this study, therefore, is to interrogate the application of tubular reactor technologies available for the production of biodiesel with a view to further escalate further research and utilization of tubular reactors for accelerated biodiesel production. Consequently, this study presents a brief overview of biodiesel feedstocks and production techniques, modes of biodiesel production using reactor technologies, and the varieties of tubular reactor technologies for the production of biodiesel. Instances of the application of tubular reactors for biodiesel production are highlighted. The outcomes of this research will enrich scholarship by providing necessary information on the types, operations, and peculiarities of various tubular reactors for biodiesel production. Refiners, researchers, and consumers of biodiesel will be better informed on the strategies and infrastructure needed for effective, economically friendly, and environmentally sustainable biodiesel production.

## 2. Feedstocks and Biodiesel Conversion Techniques

### 2.1. Feedstock for Biodiesel Production

Over the years, various techniques have been adopted for the generation of biodiesel from diverse feedstocks. Depending on the choice of feedstocks, catalysts, and costs, refiners choose the method of converting various feedstocks to biodiesel. Biodiesel is classified into generations depending on the types of feedstocks used in synthesizing them. For example, first generation biodiesel is produced from edible oils such as palm oil, olive oil, coconut oil, etc., while second generation biodiesel is generated from rubber sed oil, castor oil, jojomba oil, karanja oil, and other non-edible oils. Waste cooking oil, animal fats, recovered fats, and chicken fats are converted into third generation biodiesel. Fourth generation biodiesel is produced from algal biomass, waste cooking oil, and genetically modified biomass.

The use of edible oils for biodiesel reduces the amount of food available for human consumption, leads to high global food prices, and exacerbates food scarcity. The deployment of non-edible oils, animal fats, and waste cooking oils eliminates food vs. fuel debates and reduces the reliance on edible food crops for the production of fuel. However, non-edible oils need a large expanse of arable land, water, and time for cultivation. These not only compete with land for growing food for human consumption but also contribute to deforestation and erosion. The use of waste cooking oil, animal fats, and recovered fats allows for the conversion of wastes to fuel, does not conflict with the food chain, and serves as a sustainable means of waste disposal [6,22]. Moreover, the use of microalgae as a feedstock for biodiesel production does not conflict with the food chain, requires no fertile land and water, and has the potential to yield about 15–300 times more than other non-edible oils [23]. Fourth generation biodiesel includes photobiological solar fuels and electro-fuels. It is a novel research area where solar energy is used to convert some feedstocks into biodiesel. Such feedstocks are renewable, broadly available, inexpensive, and ecofriendly. However, the technology is not yet fully developed and requires high financial investment [24]. Table 2 shows the feedstocks, advantages, and disadvantages of the four generations of biodiesel.

### 2.2. Biodiesel Production Techniques

Biodiesel can be synthesized through two different production techniques: the physical technique and the chemical technique. The physical technique is a method of biodiesel production that does not involve any chemical reaction. This includes dilution and microemulsion. During dilution, a given volume of FB diesel fuel and other selected additives are added to natural oils to improve their viscosity and volatility. In chemical techniques, biodiesel production is achieved through the chemical modification of natural oils and fats. During the process, the physicochemical properties, and hence the behavior of the natural oils are altered. Notable examples include the pyrolysis, superfluid/ supercritical, and transesterification processes.

#### 2.2.1. Direct Use and Dilution

During dilution, more solvent is added to the solute to reduce the concentration of the solute in the solution. In physical biodiesel production, ethanol and FB diesel fuel act as the solvents for the dilution of vegetable oil. The process generates a fuel with a lower density and viscosity than vegetable oil. For example, the addition of ethanol to FB diesel fuel produces a fuel with a better combustion efficiency, an improved brake power, and brake thermal efficiency. However, the brake torque and brake specific fuel consumption of the resulting fuel is reduced. Though dilution is an easy and non-technical process, the resulting fuel suffers from incomplete combustion and more carbon deposition in the engine cylinder. Moreover, products of dilution suffer from low volatility, poor atomization, and plugging of injector nozzles [21].

#### 2.2.2. Micro Emulsion

Among the characteristics of vegetable oil which make it unsuitable as fuel for CI engine is its viscosity. The viscosity impediment of vegetable oil can be corrected by the microemulsion process. During the process, a co-solvent, alcohol, cetane improver, and surfactant are added to the vegetable oil to improve the viscosity and low liquidity. When butanol, hexanol, octanol, and methanol are added to vegetable oil or animal fats, the resultant fuel meets the optimum viscosity requirement for CI engine fuel. For example, when soybean oil was mixed with 2-octanol, methanol, cetane improver, and some surfactants (such as rhamnolipid), a clear, thermodynamically stable, and ecofriendly microemulsion biodiesel was produced. Biodiesel generated by microemulsion exhibits better cold flow properties, an enhanced stability and solubility, a lower activation energy, acceptable viscosity, and a shorter ignition delay. Moreover, the micro-emulsion of vegetable oils ensures a reduction in viscosity, a better cetane number, and improved spray characteristics in the CI engine. However, fuel synthesized by the micro-emulsification of vegetable oils and animal fats demonstrates incomplete combustion, a high deposit of carbon residue, and thickening of lubrication oils [21,24,26,27].

#### 2.2.3. Pyrolysis

Pyrolysis is a chemical method of biodiesel production during which there is the thermal decomposition of materials at elevated temperatures in the absence of air and oxygen but in an inert atmosphere. Pyrolysis can also be achieved at high temperatures (usually 400 °C–1000 °C) in the presence of a catalyst which leads to the bond formation and the coming together of small molecules. The products of pyrolyzed vegetable oils, animal fats, natural fatty acids, and methyl esters of fatty acids possess physicochemical properties, fingerprints, and characteristics similar to FB diesel fuel. Products of pyrolysis from vegetable oils, animal fats, and other feedstocks demonstrate lower viscosities, flash points, pour points, and cetane numbers when compared with FB diesel fuel. Such pyrolyzate contains satisfactory sulfur, water, and sediment contents. However, their ash content, carbon residue, and pour points are unacceptable. In addition, the high cost of infrastructure for thermal cracking, high energy cost, the use of high temperature, and problems of environmental degradation are some of the drawbacks of the process [21,26,28].

#### 2.2.4. Transesterification

**Figure 4:**
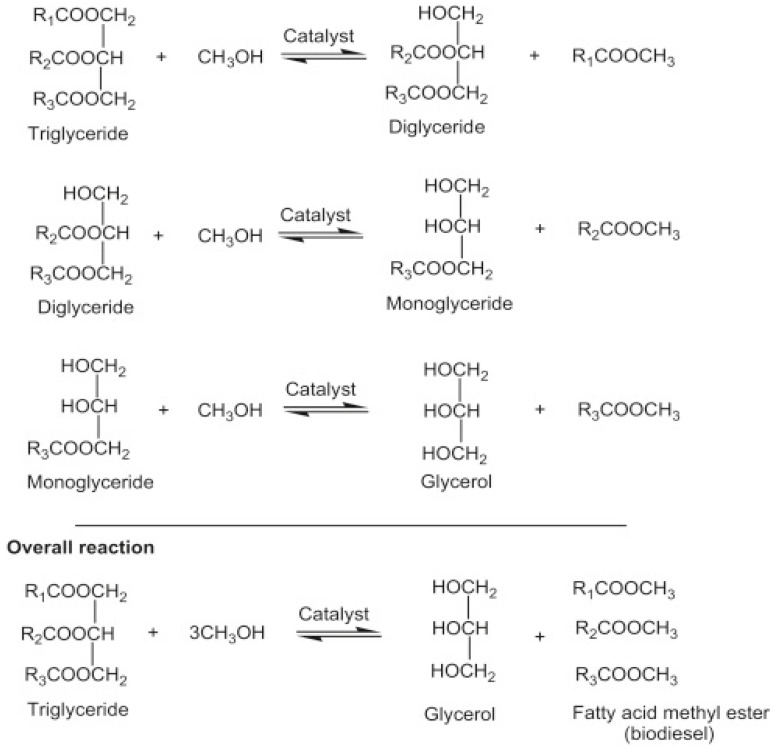
Transesterification reaction equation.

Transesterification, also known as alcoholysis, is arguably the most commonly used chemical method for converting vegetable oil, natural fats, and recovered fats into biodiesel. During the process, three moles of alcohol (methanol or ethanol) stoichiometrically react with one mole of triglyceride in the presence of a catalyst to produce mono-alkyl ester and glycerol. The three steps involved, and the general equation are depicted in Figure 4. The process occurs under moderate operating conditions of about 60–80 °C, ambient pressure, and for 90 min. Other process parameters that affect the transesterification reaction include types of catalyst, a dose of catalyst, catalyst particle size, alcohol/oil molar ratio, residence time, reaction temperature, mixing/agitation speed, choice of alcohol, and composition of oil [29].

Methanol is the more popular alcohol for transesterification owing to its higher reactivity, cheaper cost, and lower operating temperature. If methanol is used as an alcohol, the process is called methanolysis and the product is fatty acid methyl ester (FAME). If ethanol is used as an alcohol, the process is known as ethanolysis and the product is fatty acid ethyl ester (FAEE). FAEEs are less toxic and have a better cetane number, higher oxidative stability, cloud point, pour point, lubricity properties, lower iodine value, and a higher heat capacity when compared with FAMEs. However, ethanolysis is reputed for its higher cost, energy consumption, lower transesterification reactivity, higher viscosity, formation of an azeotrope with water, and formation of more stable emulsions than methanolysis. Moreover, from the ecological point of view, FAEEs emit less exhaust gas and possess a higher biodegradability in water [30,31].

In the catalytic transesterification process, the choice of catalyst greatly affects the conversion efficiency and product yield. Generally, transesterification reactions can be catalyzed by homogeneous, heterogeneous, bio-based (enzymes) catalysts, or nanocatalysts [32]. The transesterification process can be heterogeneous when the catalyst is in a different phase from the reactants and products. In this case, solid catalysts are used. However, when liquid catalysts are used, the reactants and the products are in the same phase and the process is termed homogeneous. Biobased catalysts can be either in liquid or solid phases. Table 3 compares the examples, pros, and cons of the four major classes of catalysts for the transesterification process. Though catalytic transesterification occurs at lower temperatures and has a shorter residence time, the cost of the catalyst escalates the production cost.

#### 2.2.5. Superfluid/Supercritical

The deployment of supercritical techniques for biodiesel production is one of the methods of biodiesel production and a possible substitute for the traditional biodiesel synthesis process. By definition, a supercritical fluid or superfluid is any substance existing above its critical pressure and temperature. It is a highly compressed fluid that combines the properties of both a gas and liquid. At a supercritical temperature and pressure conditions, there is no distinct liquid or gaseous phase of the substance. For example, the critical temperature and pressure of methanol, ethanol, acetone, methane, and ethane are 239.2 °C and 8.09 MPa, 240.9 °C and 6.14 MPa, 235.1 °C and 4.70 MPa, −82.6 °C and 4.60 MPa, and 32.3 °C and 4.88 MPa, respectively [46]. The use of any of these fluids under supercritical conditions greatly influences biodiesel production. Moreover, the choice of feedstock, reaction time, solvent/oil molar ratio, reactor type, and agitation speed influence the conversion efficiency of feedstock to biodiesel.

With the supercritical method of biodiesel production, there is no need for catalysts, and the process is guaranteed to produce high-quality biodiesel. When compared with other biodiesel generation methods, the supercritical technique allows for lower energy consumption. Available economic and energy analysis showed a reduction of about 71% in the cost of producing energy [47]. Notwithstanding the cost-effective and energy-efficient process, the supercritical method of biodiesel synthesis requires a high cost of production infrastructure and can denature the product. Table 4 compiles the different biodiesel production techniques and their advantages and disadvantages.

Moreover, since the spent catalyst must be removed from the product at the end of the reaction, time, energy, and water are expended during the catalyst removal process. To reduce the cost and environmental impact of commercial catalysts, researchers have turned attention to the use of waste-derived heterogeneous catalysts. Food wastes, crop residues, and agricultural wastes are now converted and developed into catalysts for biodiesel production. These efforts not only reduce the cost of production, and ensure the proper disposal of wastes, but also reduce the number of wastes at dumpsites and contribute to ecological sustainability [9,32]. At the end of the transesterification process, the spent solid catalysts are separated from the products by using a laboratory filter paper and reused. Products of the transesterification process must be purified to meet the established international standards, particularly the ASTM D6751 and EN 14214.

## 3. Modes of Biodiesel Production in Reactor Technologies

Transesterification is the most widely used method for biodiesel production. Basically, there are four steps involved in biodiesel production via transesterification. The first step is the collection of the feedstock, reagents, and other materials needed for the process. In this stage, the production reaction parameters and conditions are also determined and implemented in the reacting vessel. When the process in the reacting vessel is completed, the second step, which involves separating the slurry comprising the crude biodiesel, glycerol, catalyst, excess methanol, and other water is activated. This involves the use of the difference in densities to achieve phase separation among the resultant slurry. During this process, one of the major and predominantly low-cost gravity separation techniques including filtration, centrifugation, floatation, decantation, or sedimentation is deployed [48,49]. The heterogeneous catalyst is recovered in this stage for reuse.

In the third step, crude biodiesel is subjected to gentle heating with stirring to remove unreacted alcohol and excess moisture trapped in the biodiesel. In the fourth and final step, the biodiesel is purified to further remove any undesirable compounds such as the catalyst, soap, unconverted triglyceride, and moisture. The purification can involve the use of wet or dry washing methods, membrane filtration, and evaporation to obtain clean biodiesel. The biodiesel produced at this stage must meet the ASTM D6751 and EN 14214 standards. The wet washing purification process, though most frequently used, extends production duration, requires a large volume of clean water, and generates lots of wastewater. The treatment and disposal of wastewater and the drying of the water-washed biodiesel are energy-intensive and expensive. Drying washing is more ecofriendly, does not require water, and produces fuel of better quality when compared with wet washing. However, the cost of adsorbents and other additional apparatus makes the process uneconomical. The membrane separation technique, though still largely undeveloped and not commonly used, is environmentally benign, consumes less energy, requires no chemicals, and generates high quality products [50,51,52]. The biodiesel generation processes can be intensified by the use of reactor technologies. The deployment of reactor technologies contributes significantly to ensuring the mass production of biodiesel.

A reactor is a device or vessel with compartments where chemical reactions take place for the transformation of raw materials into desired products under specific and predetermined conditions. A reactor can also be an enclosed volume, an apparatus, or a specialized container where specific chemical reactions take place under a controlled atmosphere. A good reactor must contain mechanisms or facilities for the injection of the raw materials and other reagents, provide enough residence time for the chemical reaction to take place, and discharge the products. There must be facilities for heat addition and heat removal, safe operation and maintenance, and effective control to ensure operational safety, effectiveness, and an acceptable level of productivity. To achieve an optimum reactor operation, effective performance, and high product yield, the design stage must consider the configuration, construction materials, cost, reaction kinetics, heat and mass transfer, reaction parameters, and the environmental sustainability of the reactor [53,54]. A reactor can be operated either as a batch or a continuous process. In recent years, some researchers have reported an amalgamation of the batch and continuous process, which they dubbed the semi-batch/semi-continuous process to overcome some technical and operational associated with both batch and continuous production of biodiesel by transesterification.

### 3.1. Batch-Mode Reactors

**Figure 5:**
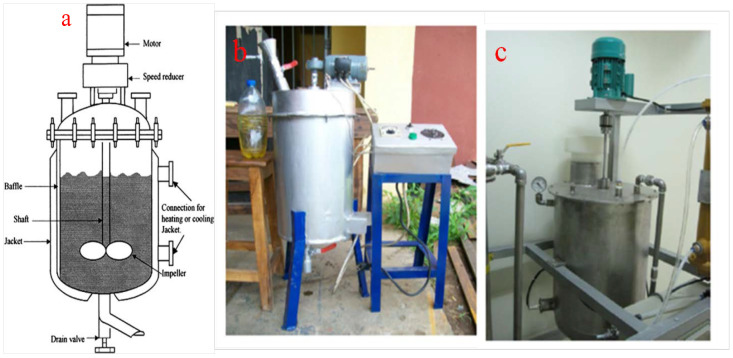
(**a**) Schematic representation of a batch-mode reactor. Fabricated batch-mode (**b**) 20 L; (**c**) 70 L reactor for biodiesel production. Adapted from [58,59].

The batch-mode reactors are the oldest, most convenient, and most popular method of biodiesel synthesis. The batch-mode reactor of biodiesel production was developed from a laboratory-scale production process by the optimization of the production parameters [16,55]. It involved the upgrading of the laboratory-scale production into commercial and industrial production scale to meet the increasing demand for biodiesel. The main feature of a batch production method is the intermittency of the process. There is no continuous flow of materials into and out of the reactor during the production period. Rather, a known quantity of raw materials is injected into the reaction and allowed to be converted into the desired product in a specified period. At the end of the process, the resultant slurry is allowed to exit the reactor and transmitted for separation, purification, and further processing [56]. When operating under the batch production mode, there is control of the inflow, adequate mixing of the reactants, and monitoring of the outflow of the materials. Despite the simplicity in the design and operation of batch reactors, the major drawbacks of the process include a longer residence time, a high operation cost, higher energy consumption, and large space requirements [57]. Figure 5 shows 20 L [58] and a 70 L [59] batch reactors for biodiesel production.

### 3.2. Semi Batch-Mode Reactors

**Figure 6:**
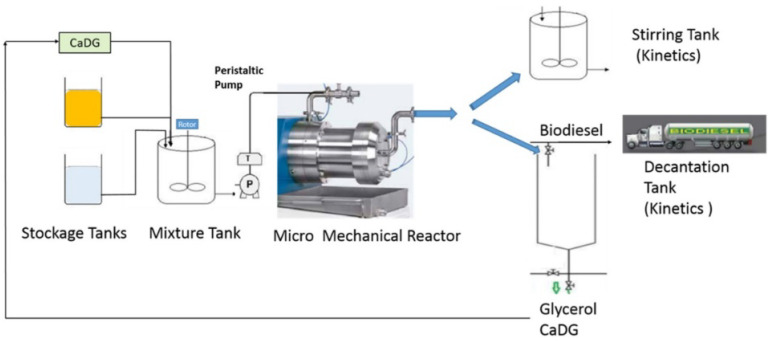

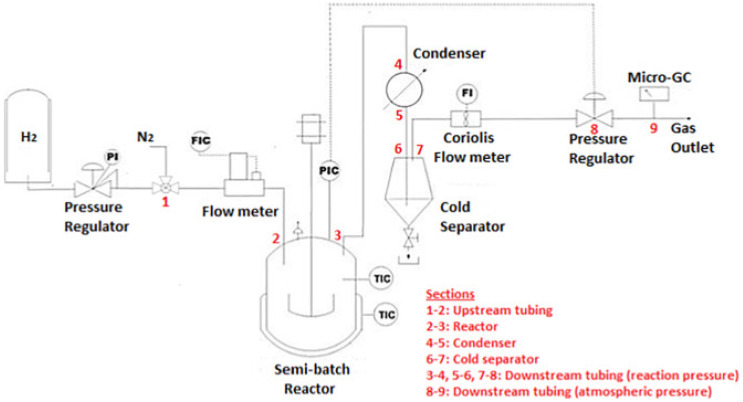
Schematic representations of a semi-continuous flow reactor for biodiesel production. Adapted from [62,63].

In the semi-batch/semi continuous mode reactor, there is the intermittent addition or removal of one more reagent or product during the process. There can also be a variation of the reaction parameters as the reaction proceeds. For example, more feedstock or methanol can be introduced into the reactor during the process to improve the reaction rate or product yield. In this way, the reaction equilibrium is altered in support of biodiesel formation by the gradual removal of the product during the process. Similar to the batch process, the semi-batch mode is characterized by a high operation cost, low production rate, and high energy consumption. There is a high rate of human intervention during the process leading to a highly strenuous and labor-intensive process [60,61,62]. Figure 6 shows the schematic diagram of a semi-continuous flow reactor for biodiesel production as reported by Malpartida et al. [63].

### 3.3. Continuous-Mode Reactors

The continuous mode reactor allows for the continuous inflow of reactants into the reactor and the simultaneous outflow of the products from the system throughout the operation period. After the initial loading of the reactor with feedstock, catalyst, and methanol, the process is initiated and agitated at the required speed to ensure a homogeneous mixing of the reactants, adequate mass, and heat transfer. At the expiration of the set residence time, the reactants are converted into products and allowed to flow out of the reactor. The process continues almost seamlessly with little or no human intervention [54,64]. The process is inbuilt with mechanisms set up to control the inflow of feedstock, catalyst, and methanol, monitoring agitation speed, residence time, and discharge of the resultant slurry.

**Figure 7:**
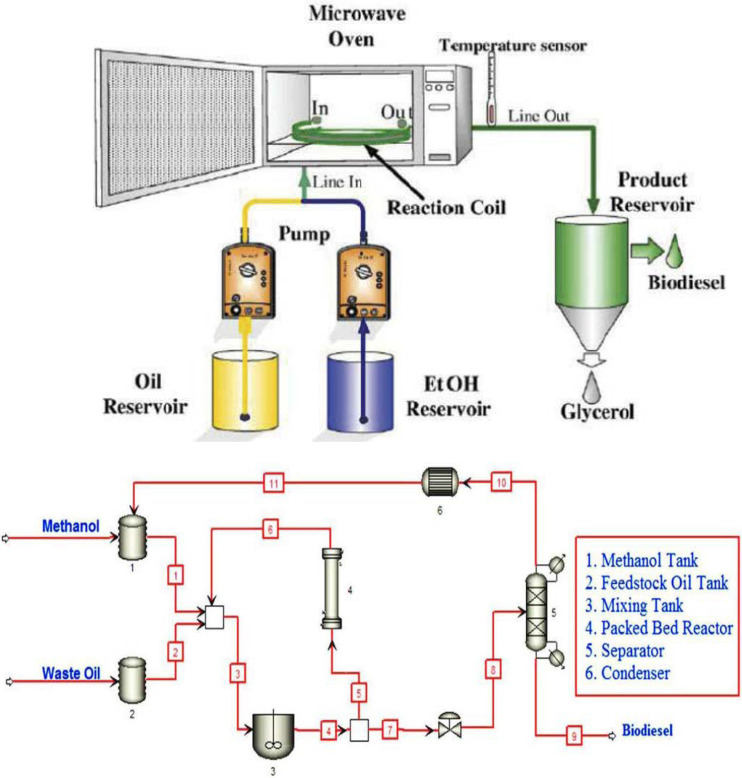
Schematic representations of a continuous flow reactor for biodiesel production. Adapted from [66,67].

It must be noted that the biodiesel production industry is moving towards a continuous mode of production and the use of automation and other innovative technologies to ensure large scale and industrial biodiesel production processes. When compared with the batch production process, the continuous production of biodiesel is achieved at lower operating costs, a reduced energy consumption, and with a less labor-intensive process [65]. The deployment of a continuous flow reactor for biodiesel synthesis increases the mass interfacial transport between methanol and oil leading to the synthesis of quality products at the lowest cost per unit volume of fuel [66]. Figure 7 shows the schematic representation of a continuous flow reactor for biodiesel production as presented by Buasri et al. [67] while Table 5 compares the three modes of the reactor operation for biodiesel production.

## 4. Tubular Reactor Technologies for Biodiesel Production

Reactor technologies for the conversion of feedstocks into biodiesel by transesterification are classified by various factors. Some of these factors include the mode of operation, operating conditions, phase numbers, mixing systems, nature of reactants and products, operating temperature and pressure, production size, residence time, mass transfer, heat exchange, and control system.

**Figure 8:**
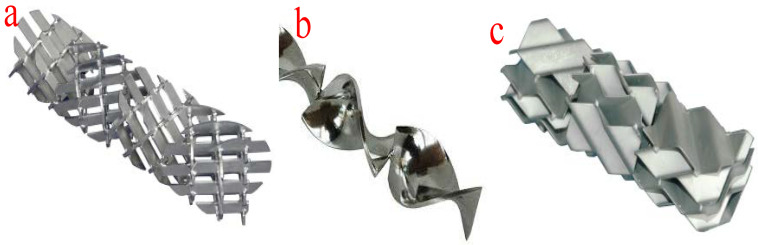
Different configurations of static mixers. (**a**) X-grid static mixer; (**b**) helical twist static mixer; (**c**) corrugated plate static mixer.

The tubular or plug-flow reactors are the simplest form of reactor technology for biodiesel production. In this type of reactor technology, reactants and reagents are fed into the reactor through the inlet and are allowed to spend some time in the reactor before being allowed to flow out from the outlet at a constant velocity. The mixing of the reactants and reagents takes place in the tubes or pipe fittings. At a constant velocity, the longer the length of the pipes, the longer the mixing time and the longer the residence time. However, the length of the mixing device and the residence time can be adjusted by altering the system pressure. Moreover, an increase in the viscosity of the mixture of the reactants and reagents will lead to laminar flow in the tubular reactors. The improvement in the reaction, length of the mixing device, and reactor size can be achieved by deploying various mixers such as in-line mechanical mixers, static mixers, and other injection devices. Moreover, the application of static mixers ensures effective radial mixing of multiple immiscible flowing liquids. Figure 8 shows the different configurations of static mixers. The suitability of a typical static mixer is determined by the type of reaction, reaction temperature, reactor configuration, Reynolds number, and viscosity of the fluids. These configurations facilitate the efficient transesterification of the different oils used as feedstock.

When compared with other reactor technologies, tubular reactors are more efficient, require minimum maintenance, and ensure the fast and homogeneous mixing of the fluids. They are not capital intensive and require less space for the construction. Reactors operating on the tubular technology can be used at high pressure and under steady-state conditions. The reactor technology allows continuous operation over a long period and easy product separation. This ensures adequate product separation and recovery of excess methanol and unreacted oils for recycling. Moreover, there is a short residence time when using tubular reactors due to the reduced length of the reactor [68,69]. However, there is a noticeable temperature and pressure drop during the reaction and between the inlet and the outlet points. Moreover, these reactors experience significant temperature changes at different points between the inlet and outlet. Moreover, the reactor requires a large length-to-diameter ratio and a limitation for the Reynolds number. In most cases, tubular reactors require a slow mixing process which often leads to large hold-ups and clogging [21,70]. Notable examples of tubular/plug flow reactors include packed bed reactors, fluidized bed reactors, trickle bed reactors, oscillatory flow reactors, and micro-channel reactors.

### 4.1. Packed Bed Reactors

**Figure 9:**
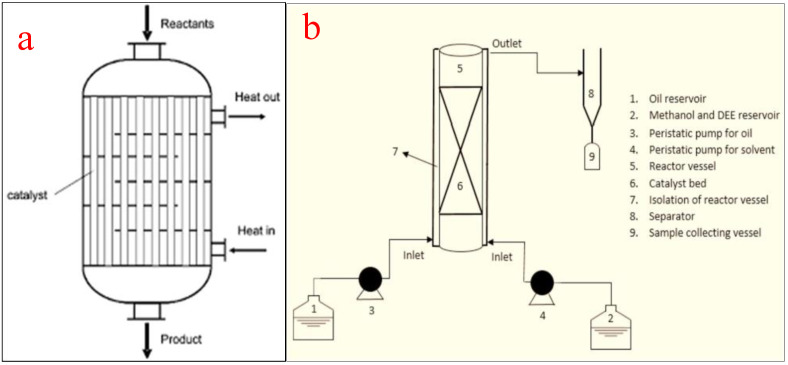
Packed bed reactor. (**a**) schematic representation; (**b**) a packed bed reactor for biodiesel production. Adapted from [72].

Packed bed reactors, also known as fixed bed reactors, are one of the most used tubular reactors in chemical industries, especially for biodiesel production using heterogeneous catalysts. They can also work in the supercritical mode for improved biodiesel production. During the transesterification process for biodiesel production, the packed bed reactor provides a substrate for enzyme immobilization to improve production. Much more than the size or volume of the reactor, the amount of the solid catalyst in the tube influences the conversion of the feedstock into biodiesel [21]. The reactors are in tubular forms and the tubes are filled with packing materials including heterogeneous catalysts and activated carbon. The performance of a packed bed reactor is greatly affected by the catalyst particle size, bed structure, and the spaces between catalyst particles. The arrangement of the packing materials is governed by factors such as (i) physical attributes of the tube, (ii) the shape, size, and the surface structure of the catalyst, and (iii) the intensity, method, and speed of deposition [71]. Figure 9a shows the schematic representation of a packed bed reactor while (b) shows a packed bed reactor for biodiesel production [72].

With packed bed reactors, the higher conversion efficiency of oils per unit of solid catalysts is feasible and shortens the residence time. Another major benefit of using packed bed reactors is the downstream elimination of catalysts from the product since the catalysts are packed in the tube. According to Sakdasri et al. [73], the greatest advantage of the deployment of packed bed reactors is their high conversion efficiency and ability to use heterogeneous catalysts. Despite these advantages, the reactor suffers from acute and sudden pressure drops, increased cost of operation, and high energy consumption. The pressure drops can be attributed to fluid friction, fluid viscosity, and reactor tube length. Because of these advantages, several researchers have utilized packed bed reactors for biodiesel production.

### 4.2. Fluidized Bed Reactors

**Figure 10:**
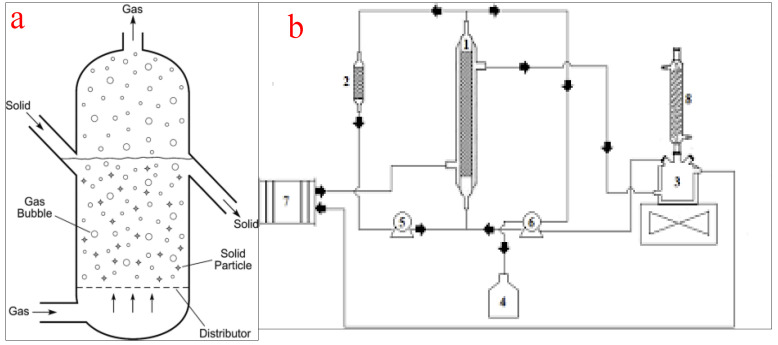
(**a**) schematic representation of a fluidized bed reactor; (**b**) a typical fluidized bed reactor for biodiesel production. (1 = reactor; 2 = reactor column; 3 = substrate reservoir; 4 = product vessel; 5 and 6 = peristaltic pumps; 7 = thermostatic bath; 8 = reflux condenser). Adapted from [75].

Fluidized bed reactors, also known as expanded bed reactors, are the most popular configurations employed for the conversion of oils into biodiesel on a laboratory or commercial scale. The basic principle of the operation of a fluidized bed reactor involves a fluidization medium (gas or liquid) made to flow through the bed of solid reactants at a velocity high enough to suspend the solid and make it behave as a fluid. The reactor consists of a reservoir and a column. The reservoir is for the housing and preparation of the liquid feedstock while the column consists of a calming section, distributor, fluidized bed, and freeboard. The calming section helps to equalize the liquid feedstock flow while the distributor creates enough pressure difference across the fluidized bed. At a low fluid velocity, the particle in the vessel is stagnant, similar to the packed bed reactors. However, as the fluid velocity increases, the drag force will overcome the weight of the fluid and propel the particles into an upward movement which signifies the start of the fluidization process. At a higher fluid velocity, the particles expand and swirl around and upward in the fluidized bed. The freeboard disallows the catalyst from flowing out of the column [21,74]. Figure 10a shows the schematic representation of a packed bed reactor while (b) shows a packed bed reactor for biodiesel production as used by the authors of [75].

Fluidized bed reactors have become popular for the transesterification of oil into biodiesel due to their ability to ensure uniform particle mixing, uniform temperature gradients, and the ability to be operated effectively on a continuous scale [76]. However, the sudden pressure loss in the column creates a pressure loss scenario and the possible erosion of internal components. Moreover, due to the likely expansion of the bed materials in the reactor, there is a need for an increment in the reactor size and consequently, the cost of the reactor construction. Other disadvantages of fluidized bed reactors include a high operating cost, reactor wall erosion, the likelihood of particle entrainment, and high catalyst attrition [77]. The practical application of a fluidized bed reactor by Kutálek et al. [78], Fidalgo et al. [75], and Wang et al. [79] yielded a biodiesel conversion efficiency of 77%, 98.1%, and 91.5% respectively.

### 4.3. Trickle Bed Reactors

Trickle bed reactors are some of the most used industrial reactors in chemical and related industries including the electrochemical, petroleum, petrochemical, coal, pharmaceutical, oil and gas, waste treatment, and biochemical processes. A notable application of trickle bed reactors includes the conversion of vegetable oil into biodiesel, hydrogenation of biooils, polymerization of monomers, purification of feedstocks, and manufacturing of pharmaceuticals [80]. It is a continuous system where liquids are made to flow through a packed bed containing a packing medium. There is a platform for the solid, liquid, and gas based on gravity or pressure forces.

**Figure 11:**
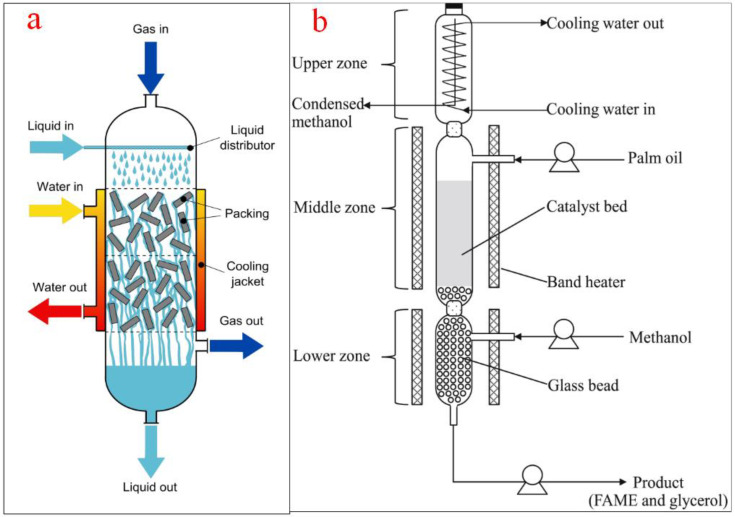
(**a**) Schematic representation of a trickle bed reactor; (**b**) a typical trickle bed reactor for biodiesel production. Adapted from [83].

The trickle bed reactors for biodiesel production consist of a tubular tank and structure for solid catalysts at the base of the reactor [81]. The feedstock is introduced from the top of the column while the alcohol can be fed either top or bottom. The heating jacket mounted at the reactor wall helps to maintain the reaction temperature. The continuous heating ensures that the alcohol is vaporized while the unreacted alcohol can be separated from the product. The outlet at the top of the reactor allows for alcohol gas recycling while the outlet at the bottom of the reactor is for the products and unreacted oil to flow out [82]. Figure 11a shows the schematic representation of a trickle bed reactor while Figure 11b shows a typical trickle bed reactor for biodiesel production as used by Jindapon et al. [83].

Major advantages of using trickle bed reactors include simplicity in operation even at a high temperature and pressure, high catalyst loading per unit volume, and low capital and operating costs. Moreover, trickle bed reactors can be used for diverse applications and can accommodate a large volume of production. When used for biodiesel production, trickle bed reactors ensure a higher feedstock conversion rate and improve product productivity [81]. Despite these benefits, trickle bed reactors suffer from poor heat transfer rate, limited diffusion among particles, and unequal fluid distribution. Trickle bed reactors are difficult to scale up and controlling vessel parameters might pose a huge challenge [84]. In research, Muharam et al. [80] reported a 78.22% conversion efficiency while Jindapon et al. [83] reported a biodiesel yield of 92.3% and a product purity of 93.6%.

### 4.4. Oscillatory Bed Reactors

This type of tubular reactor contains equally spaced tubes with orifice plate constrictions arranged to generate oscillatory flow with intermittent changes in the flow direction using a piston drive. This unique oscillation motion produces vortex mixing that results in the filling of the entire cross-section of the baffles cavity due to fluid obstruction. The configuration of the baffles, rather than the Reynolds number of the fluid, plays an important role in the effectiveness of the reactor. Typically, baffles can be of helical, axial, integral, or wire wool configurations with a tube diameter of less than 15 mm to ensure vigorous mixing and to minimize frictional loss. For the purpose of biodiesel production from vegetable oil using heterogeneous catalysts, a tube diameter of about 5 mm is recommended to minimize the overbearing construction and feedstock costs. In the same vein, an oscillatory Reynolds number of 10 is adequate to ensure turbulent flow in the tube [85].

**Figure 12:**
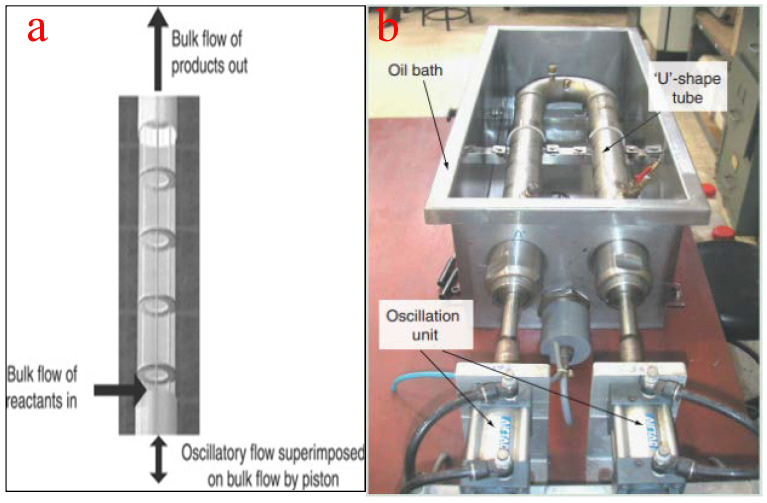
(**a**) Schematic representation of an oscillatory flow reactor; (**b**) a typical oscillatory flow reactor for biodiesel production. Adapted from [88].

The use of oscillatory flow reactors ameliorates the challenges associated with the deployment of conventional flow reactors by ensuring vigorous mixing, superb heat transfer, and an excellent plug flow experience. The flow generated by the oscillatory motion is not affected by the net flow rate, the residence time, and the hydrodynamic properties of the slurry. Similarly, the moderation of the net flow rate ensures a smaller reactor volume, a compact setup, minimizes space requirement, and guarantees quality mixing [86]. To achieve efficient and economically viable biodiesel production, oscillatory flow reactors should have a short length/diameter ratio [87]. Figure 12a depicts the schematic representation of an oscillatory flow reactor while Figure 12b shows a typical oscillatory flow for biodiesel production as reported by Masngut et al. [88].

When compared with conventional reactors, oscillatory flow reactors consume less energy and generate less waste. They also offer improved mixing efficiency and better mass and heat transfer. The reactor can be operated on both baths and continuous modes offer process flexibility and can easily be scaled up to accommodate increased production [89]. However, the oscillatory flow reactor suffers acute pressure drops as a result of persistent frictional loss. The generation of gas bubbles during operation dampens the oscillations and upsets the plug flow [90].

### 4.5. Micro-Channel Reactors

Micro-channel reactors are another type of tubular reactor using micro-channel technology for processing chemicals and other diverse applications. They are made up of narrow channels or tubes, in the millimeter range, which allow a high surface/volume ratio, minimize diffusion length, and improve mass and heat transfer. The flow of fluid in this type of reactor is orderly, predictable, and measurable, which also requires a lengthy pipe to ensure thorough mixing [91]. The requirement of a lengthy mixing path is a challenge that is addressed by the application of passive micromixers. The deployment of micromixers of diverse configurations and arrangements achieves an improved contact surface through the mixing of two or more liquids. For example, serial lamination micromixers split the inlet flow and merge them first horizontally and then vertically. For injection micromixers, the oil is allowed to split into substreams before the injection of methanol through a collection of nozzles. Droplet micromixers employ an internal flow field to ensure the mixing of the liquids and transport them by capillary effects, pressure gradient, and flow instability of two or more fluids [92,93].

**Figure 13:**
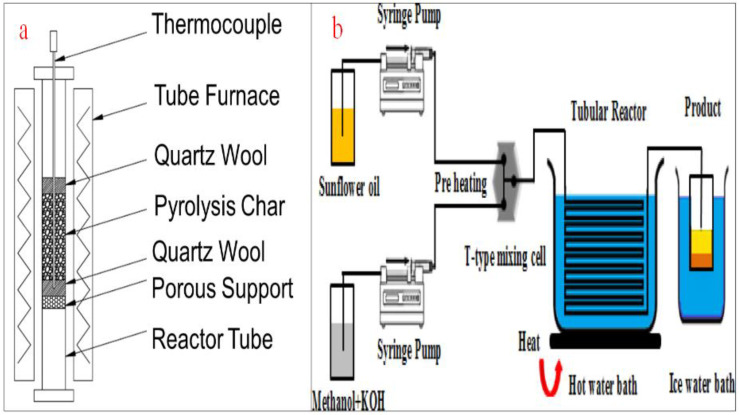
(**a**) Schematic representation of a micro channel reactor; (**b**) a micro channel for biodiesel production. Adapted from [97].

When compared with conventional reactors, micro-channel reactors demonstrate a high surface/volume ratio, better heat and mass transfer, and improved homogeneous fluid mixing. This type of reactor also allows a shorter reaction duration, less degradation, better scalability, and easier optimization and monitoring. With micro-channel reactors, there is an opportunity for more precision reaction control, selectivities, better conversion efficiency, faster reaction speed, and improved product yield. Moreover, temperature control is easier and more precise, safer, and allows for prompt phase separation [94,95]. However, the micro-channel reactors can handle a limited volume of feedstock at a time due to the small volume of the tubes. They are also prone to intermittent clogging, fouling, tube blockage, and corrosion. Other drawbacks of this type of reactor include a high rate of leakages between channels and the prohibitive cost of building the reactor. Because the micro-channel reactors are small, they are not usually applied at an industrial scale [64,96]. Figure 13a depicts the schematic representation of a micro channel reactor while Figure 13b shows a typical micro channel reactor for biodiesel production as reported by Baydir and Aras [97].

Generally, tubular reactors are some of the simplest and easy to construct and operate chemical reactors for biodiesel production. They are cost effective, environmentally friendly, and safe to operate. They can be operated both on batch and continuous modes, are easy to clean, and ensure a high product yield. The quality of the product generated by tubular reactors meets international standards. Though some of them suffer from sudden pressure drops, and high catalyst attrition, they nonetheless find practical industrial applications. Table 6 shows the major benefits and drawbacks of tubular reactors.

## 5. Recent Applications of Tubular Reactors for Biodiesel Production

Biodiesel production has been intensified through the application of reactors to meet the ever-growing demand for quality biodiesel. The use of glassware in the laboratories has not only proved inadequate and time-wasting but also not replicable in most cases. The quality of the product cannot be guaranteed after each production cycle due to many factors. Therefore, to ensure uniformity in standards, increased production efficiency, and cost-efficient production, there is critical need to adopt some proven intensification processes [54]. The use of reactors is one of the biodiesel intensification processes. For the industrial and mass production of biodiesel, the use of tubular or plug flow reactors has been massively exploited. The application of tubular reactor technologies for biodiesel production ensures cost effectiveness, improves the production rate, and allows the use of innovative technologies. Though the initial financial investment in reactor construction might be daunting, in the long run, the cost per liter is significantly lower than laboratory-scale production. There are opportunities for scaling-up, process optimization, reproducibility, quality assurance, and minimum human intervention with tubular reactor technologies [57,94]. Table 7 shows the compilation of the deployment of tubular reactor technologies for the production of biodiesel.

In recent research, Zik et al. [104] deployed a packed bed reactor to produce biodiesel from waste cooking oil (WCO) using CaO derived from chicken bones as a catalyst. Working at a reaction temperature of 65 °C, methanol:oil ratio of 6:1, and a mixing speed of 600 rpm, a biodiesel yield of 98.4% was recorded. In another study, Hashemzadeh Gargari and Sadrameli [72] used a packed bed reactor to transesterify linseed oil into biodiesel in the presence of methanol and CaO. The reaction yielded 98% biodiesel of acceptable quality and compatible with ASTM D 6751 and EN 14214 standards. Similarly, Talha and Sulaiman [105], Ani et al. [106], Akkarawatkhoosith et al. [107], and Jindapon et al. [108] recorded 95%, 72.5%, 99%, and 95% when a packed bed reactor was used to convert various feedstocks into quality biodiesel. The authors were unanimous in affirming the ease of operation, moderate operating conditions, high product yield, and operational advantages derived from the use of a packed bed reactor for effective biodiesel production.

The use of a fluidized bed reactor for biodiesel production has been investigated and found to be operationally feasible by various researchers in recent times. Using a magnetic whole-cell biocatalyst, Chen et al. [109] converted pretreated WCO into quality biodiesel in a novel magnetically fluidized bed reactor with a column internal diameter of 100 mm and 950 mm length. With a reaction temperature of 35 °C and a catalyst concentration of 12 wt%, a biodiesel yield of 91.8% was achieved. In another study, Zhou et al. [110] utilized a fluidized bed reactor to synthesize soybean oil into biodiesel using a magnetic chitosan microspheres-immobilized lipase as a catalyst. The reactor consisted of a glass column of 30 mm inner diameter and 400 mm height maintained at 35 °C for 72 h and a 25 mL/min fluid flow rate. At the end of the reaction, a biodiesel yield of 82% was recorded. A product yield of 98.1% and simultaneous glycerol separation and removal were recorded when a 42.4 cm^3^ glass tube fluidized bed reactor was deployed for the transesterification of babassu oil into biodiesel [75]. The generated biodiesel was of good quality and in line with international standards. Similar results were recorded by Liu et al. [111] when methanol and 16 wt% magnetic whole-cell biocatalysts were used to transesterify waste frying oil into biodiesel in a fluidized bed reactor. The results of these investigations confirm the feasibility of a fluidized bed reactor to improve biodiesel yield, glycerol removal, and catalyst stability in tolerable reaction conditions. The generated biodiesel was of acceptable quality and complied with the ASTM D6751 and EN 14214 standards.

A 91 mL thermally insulated trickle bed reactor was applied for the production of biodiesel from rapeseed oil, methanol, and a heterogeneous Ca/Al oxide composite catalyst. The transesterification process progressed significantly well in the reactor leading to simultaneous biodiesel and glycerol separation and collection with over 94% biodiesel yield [112]. Moreover, Son and Kusakabe [113] used a trickle bed reactor (internal diameter = 16 mm, height = 200 mm) for the conversion of low-grade used sunflower oil into biodiesel using CaO as a catalyst. The authors reported a 98% biodiesel yield and the continuous separation of excess methanol and glycerol from the product. Jindapon et al. [83] achieved a 92.3% product yield when methanol and calcined dolomitic rock were used as raw materials to transesterify palm oil to quality biodiesel in a trickle bed reactor (glass column with an internal diameter of 3 cm and 40 cm long). The authors listed high biodiesel yield, better glycerol quality, recovery of excess methanol, and removal of glycerol as the novel benefits of the process.

Using a 15 L cylindrical oscillatory flow reactor, NaOH catalyst, and methanol, García-Martín et al. [114] converted WCO into biodiesel and tested the fuel in a 140 hp EURO4 test bed engine. The easy to operate and energy-efficient reactor achieved 72.5% product yield, easy separation of glycerol from the product, and the generated fuel met ASTM D6751 and EN 14214 standards. In a similar vein, Kefas et al. [115] deployed an oscillatory flow reactor to convert palm fatty acid distillate and modified sulfonated glucose catalyst into biodiesel using methanol as alcohol. At the end of 50 min residence time and a reaction temperature of 60 °C, a biodiesel yield and conversion efficiency of 94.21% and 97.1%, respectively, were achieved. The same pattern of results was obtained when Soufi et al. [116], Phan et al. [117], and Santikunaporn et al. [118] achieved 81.9%, 97%, and 99.7%, respectively, when they engaged an oscillatory flow reactor to generate biodiesel from vegetable oils. The authors listed an enhanced product yield, uniform mixing, low and uniform shear, improved heat and mass transfer, compact reactor design, and linear scalability as some of the benefits of using oscillatory flow reactors for biodiesel production [90].

The use of a microchannel reactor for biodiesel generation has been investigated by various researchers. Mohammadi et al. [119] used soybean oil, 5 wt.% calcined CaO, and methanol (methanol:oil ration of 12:1), at 65 °C for 5 min. The reactor had a 52% biodiesel yield after 5 min, which was a significant improvement from the conventional reactor. Wen et al. [120] used a 316 L stainless steel micro-channel reactor to generate methyl ester from soybean oil using NaOH and methanol. The process was maintained at 56 °C and a biodiesel yield of 99.5% was achieved after 28 s. This is a significant improvement over conventional reactors, in terms of short residence time and product yield. The works of Azam et al. [121], Dai et al. [103], and Kaewchada et al. [122] in achieving biodiesel yields of 100%, 99.5%, and 97.14%, respectively, attest to the viability of the micro-channel reaction for biodiesel production.

## 6. Chemical Kinetics of Biodiesel Production by a Tubular Reactor

The tubular reactor is the simplest reactor to achieve the transesterification of triglycerides to biodiesel [123]. Transesterification is a slowly reversible reaction. The forward reaction is achieved with a lower activation energy than the backward reaction, and therefore favors the conversion of feedstock to biodiesel [124]. As shown by the reactions in Figure 4, the two intermediate compounds formed during transesterification are diglycerides (DG) and monoglycerides (MG). The final products, i.e., biodiesel and glycerol (GL) are generated during the third stage due to the consecutive reversible reaction involved in the transesterification of triglyceride (TG) to biodiesel. However, many factors can contribute to reducing the activation energy, influencing the forward reaction, and consequently increasing product formation.

The transesterification reaction is greatly influenced by the quantity of alcohol present in the reactor. Raheem et al. [125] reported that excess methanol in the reacting chamber enhances the speedy conversion of DG and MG to FAME particularly when the ratio of methanol to oil exceeds the stoichiometric value. The chemical kinetic study of transesterification is a process of three orders. The compound of the second order chemical kinetic of transesterification is developed from the first order. Equations (1)–(3) show the kinetics of the chemical process [125]:

I_1_ = *k*_1_ C_TG_·C_MOH_ − *k*_−1_ C_DG_·C_FAME_ (1)

I_2_ = *k*_2_ C_DG_·C_MOH_ − *k*_−2_ C_MG_·C_FAME_ (2)

I_3_ = *k*_3_ C_MG_·C_MOH_ − *k*_−3_ C_GL_·C_FAME_ (3)

where I = Specific reaction rate (mol g^−1^ min^−1^),

C = Concentration (mol^−1^),

*k*_i_ = Reaction rate constant forward reaction (I_2_ mol^−1^ min^−1^ g^−1^),

*k*_−i_ = Reaction rate constant for reverse reaction (I_2_ mol^−1^ min^−1^ g^−1^)

The conversion of Equations (1)–(3) into yields Equations (4)–(6), respectively.

(4) $$-\frac{d\left[TG\right]}{dt}=k_{1}\left[TG\right]\left[CH_{3}OH\right]-k_{-1}\left[DG\right]\left[FAME\right]$$

(5) $$-\frac{d\left[DG\right]}{dt}=k_{2}\left[DG\right]\left[CH_{3}OH\right]-k_{-2}\left[MG\right]\left[FAME\right]-k_{1}\left[TG\right]\left[CH_{3}OH\right]-k_{-1}\left[DG\right]\left[FAME\right]$$

(6) $$-\frac{d\left[MG\right]}{dt}=k_{3}\left[MG\right]\left[CH_{3}OH\right]-k_{-3}\left[GL\right]\left[FAME\right]-k_{2}\left[DG\right]\left[CH_{3}OH\right]-k_{-2}\left[MG\right]\left[FAME\right]$$

The combination of Equations (4)–(6) yields Equation (7):

(7) $$K_{1}=\frac{k_{1}}{k_{-1}}$$

(8) $$K_{2}=\frac{k_{2}}{k_{-2}}$$

(9) $$K_{3}=\frac{k_{3}}{k_{-3}}$$

The first order chemical kinetic design model measures the effect of time and reaction temperature on the conversion process. With the negligible impacts of the catalyst concentration, the first step of the process is assumed to be the forward reaction only while the backward reaction is neglected. The conversion rate for the irreversible first order kinetics is shown in Equation (10). When t = 0, ln[TG]_0_ = 0 but as the [TG]_0_ approaches 1.0 mol/dm^3^, the [TG]_t_ is the percentage of biodiesel yield at time, t. The conversion of TG to FAME is defined as X_FAME_ (Equation (11)) [125]:

(10) $$-r_{a}=\frac{-d\left[TG\right]}{dt}=k\left[TG\right]\times {\left[CH_{3}OH\right]}^{3}$$

(11) $$X_{FAME}=1-\frac{\left[TG\right]}{{\left[TG\right]}_{0}}$$

The irreversible second order chemical kinetic model gives the conversion rate as shown in Equations (12) and (13), with k as the rate constant.

(12) $$-r_{A}=\frac{-d\left[TG\right]}{dt}=k\times {\left[TG\right]}^{2}$$

(13) $$\frac{dX_{FAME}}{dt}=k{\left[TG\right]}_{0}{\left(1-X_{FAME}\right)}^{2}$$

The reversible second order chemical kinetic model ensures there is adequate collision and reactions between the reactants in the overall reaction process for the continuous production of biodiesel. These collisions must be at the required process conditions. The combination of Equations (14) and (15) helps to achieve Equation (16) [126,127].

(14) $$\left[TG\right]=\left({\left[TG\right]}_{0}-X_{FAME}\right)\;\mathrm{and}\;\left[CH_{3}OH\right]=\left[CH_{3}OH\right]-X_{FAME}$$

(15) $$\frac{d\left[X_{FAME}\right]}{dt}=\frac{k_{1}k_{3}}{k_{2}}\left({\left[TG\right]}_{0}\right)g(\left[CH_{3}OH\right]-X_{FAME}$$

(16) $$y\left(t\right)=\frac{1}{{\left[CH_{3}OH\right]}_{0}-{\left[TG\right]}_{0}}ln\frac{{\left[TG\right]}_{0}{\left[CH_{3}OH\right]}_{0}-X_{FAME}}{{\left[CH_{3}OH\right]}_{0}{\left[TG\right]}_{0}-X_{FAME}}=\frac{k_{1}k_{3}}{k_{2}}t$$

Generally, a higher temperature increases the appropriate energy required to ensure a more effective collision among the reacting molecules and ensure the digestibility of the reactants. In a tubular reactor for biodiesel production, the tubes must be properly lagged and none of the reactants must be exhausted. Designing and understanding the chemical kinetics that govern the transesterification reaction will accelerate biodiesel production, especially when combined with the use of tubular reactor technologies.

## 7. Implications and Future Perspectives

While it is incontrovertible that the deployment of a tubular reactor will escalate the production of biodiesel, the impact of such an increment should not be lost. Undoubtedly, the use of tubular reactor technologies will ensure the democratization of biodiesel production and utilization, popularize the use of biodiesel for diverse applications, allow countries to move away from the use of FB fuels, create employment and other socioeconomic benefits, and ultimately slow down environmental degradation [128]. However, there are direct and indirect impacts of the deployment of tubular reactor technologies for the acceleration of biodiesel production on various aspects of our lives and environment.

Acceleration of biodiesel production will increase the use of biodiesel as internal combustion engine fuel thereby improving engine performance and mitigating the emission of CHGs and the environmental pollution associated with FB fuels. The use of waste cooking oil, waste animal fats, recovered kitchen fats, and other forms of wastes as feedstock for biodiesel production will improve sanitation, contribute to waste management, and reduce the contamination of aquatic and terrestrial habitats. Converting these wastes to biodiesel will also improve air quality, eliminate unpleasant odors, prevent the breeding of flies and other disease-causing pathogens, and improve human health [129,130]. Acceleration of production and utilization of biodiesel will, however, increase the emission of NOx from internal combustion engine tailpipes. There will also be increased water utilization for biodiesel purification.

Though the cost per liter might reduce, the cost of capital investment will skyrocket as a result of the deployment of tubular reactor technologies for the acceleration of biodiesel generation. The total cost of biodiesel production includes the cost of raw materials, cost of the plant, labor costs, cost of energy, etc. These are dependent of the production scale, production technique, type of raw materials chosen, and location of the plant, among others [131]. The cost of raw materials (feedstock, catalyst, chemicals, etc.) accounts for about 70–80% of the total production cost of biodiesel. However, with the utilization of used vegetable oil and waste animal fats as feedstock and the use of waste-derived catalysts which are reusable for many production cycles, the cost of raw materials has been significantly reduced. For example, the plant cost, consisting of reactor purchasing, land acquisition, piping, instrumentation, installation, and electrical and auxiliary facilities requires huge financial commitments and is second only to the cost of raw materials. Bokhari et al. [132] and Karmee et al. [133] agreed that adapting biodiesel intensification approaches will escalate the cost of production.

Other implications of tubular reactor technologies for biodiesel production are the anticipated increment in net GHGs from land-use change, deforestation, food security, water scarcity, and resource utilization. Though the tubular reactor ensures the quick and effective mixing of fluids, a high product yield, and better heat and mass transfer, there are legitimate concerns about process control and sustainability criteria. The increased need to use a reactor for biodiesel production is to reduce the production cost, energy and water requirements, reaction times, human intervention, and labor costs. The current ecological burden, unpredictable product quality, lack of ability to meet ASTM and EN standards, low conversion efficiency, and safety concerns associated with laboratory-scale biodiesel production will be addressed with industrial-scale production.

To meet the expected market share of biodiesel, feedstock price, availability, volatility, and accessibility are major factors. Feedstock that does not affect food change, nor requires land and water should be developed, tested, and nurtured to maturity to guarantee massive biodiesel production. Such feedstock should be subjected to effective pretreatment techniques to aid its digestibility and improve the conversion efficiency [134]. Compact biodiesel production plants with less energy consumption, and a minimum carbon footprint but using innovative technologies are needed. Such plants must require minimum human intervention and rely on robotics technology, artificial intelligence, and smart metering. Novel manufacturing techniques and practices must be developed to construct state-of-the-art reactors for biodiesel production.

Going forward, more investigations are needed to discover more advanced transesterification reactors with the capability to convert used vegetable oil, animal fats, and natural oils into biodiesel. The use of locally available construction materials and methods for reactors should be encouraged. Innovative, economic, and eco-friendly technologies must be embraced to replace conventional methods with a view to improving the sustainability of the process. The selection of appropriate, locally available, low-cost, and high-yielding feedstock is one of the most crucial criteria for sustainable and large-scale biodiesel production. There must be deliberate efforts for the targeted sensitization of the populace to improve the acceptability of biodiesel. The identified factors responsible for negative attitudes and other social repellents of biodiesel among people from diverse socioeconomic statuses must be addressed.

## 8. Conclusions

The use of tubular reactor technology for the intensification of biodiesel production is key to ensuring improved production, commercialization, and the better-quality production of biodiesel. The increased production of biodiesel will reduce the challenges associated with the adaptation and utilization of biodiesel for diverse applications. The commercialization, increased production, and utilization of biodiesel will benefit the environment, assist in the diversification of the fuel base, provide viable alternatives to FB fuels, limit the emission of GHGs, and slow down environmental pollution. The deployment of the tubular reactor for biodiesel production will create employment, ensure environmental cleanliness, prevent contamination of surface and underground water bodies, and increase the utilization of the quality of biodiesel for diverse applications.

The production of biodiesel must be incentivized through provision subsidies, and tax exemptions to encourage biodiesel refiners. Land must be made available for investors to build industrial-scale tubular reactors to ensure the commercial generation of biodiesel. More wastes must be brought into the feedstock basket to further bring down the cost of raw materials for biodiesel production. The large-scale generation of biodiesel under a circular economy must be escalated to enjoy the technical, sanitary, socioeconomic, and environmental benefits associated with the application of reactor technologies for biodiesel generation. The use of innovative technologies such as robots, smart metering, artificial intelligence, machine learning, genetic algorithms, cloud computing, smart cameras, and other modeling tools must be introduced into biodiesel research.

## Figures and Tables

**Table 1:** Benefits and drawbacks of biodiesel [9,10,11].

Criteria	Benefits	Drawbacks
Renewability	Renewable and biodegradable	
Safety	Safe and non-toxic	
Environment	Ecofriendly Environmentally sustainable	
Storage	Safer to handle, store, and transport	Can deteriorate in storage
	Compatible with FB fuel storage facilities	
Properties	High energy content	High viscosity
	Low sulfur content	High pour point
	High cetane number	
	High flash point	
Performance	Performs better than FB diesel fuel	High fuel consumption
	Contributes to power generation	Clogging of fuel filter and fuel lines
	Better thermal efficiency	
	Lower noise level	
Emission	Emits less carbon and other GHGs	Emits more NOx
	High combustion efficiency in ICEs	
	Lower smoke generation	
Combustion	Improved combustion in ICEs	Low cylinder pressure
	Better combustion speed	Reduction in heat release
Feedstocks	Readily available and low-cost feedstock	Some of the feedstocks conflict with food supply
	Synthesized from renewable feedstocks	Some feedstocks need to be cultivated
	Conversion of wastes to fuel	
Economy	Reduces fuel importation and saves foreign exchange	
	Contributes to economic growth and environmental sustainability	
	Employment generation along the value chain	
Application	Can be used without engine modifications	Unsuitable for cold temperature regions
	Contributes to power generation	Can harm rubber hoses in engines
Production	Can be produced locally by households	Unpredictable standards

**Table 2:** Examples of feedstocks for the four generations of biodiesel [6,24,25].

Generations of Biodiesel	Feedstocks		Advantages	Disadvantages
	Types	Examples		
First	Edible oils	Coconut oil Palm oil Corn oil Olive oil Mustard oil Sunflower Rice bran Rapeseed oil Hazelnut oil	Readily available Simple conversion process Safe handling and transportation Easily adaptable to existing infrastructure Easy to mix with FB diesel fuel	Affect food security Initiate food vs. fuel debate Rising food costs Cultivation of feedstocks requires land and time Shortage of arable land for cultivation
Second	Nonedible oils	Rubber seed oil Sapindus oil Mukorossi oil Thevettia peruviana oil Jatropha curcus Jojoba oil Karanja oil Neem oil Mahua indica oil	Do not affect the food supply Cheap feedstock Seed, grains, and residues are used as feedstock Low conversion cost Readily available Generation of other products Ecofriendly	Large expanse of land and water needed to grow feedstock Underdeveloped conversion technologies Complicated production processes Induce soil degradation, erosion, deforestation, and bush burning
Third	Waste oils algae	Animal tallow Chicken fat Poultry fat Recovered fat Fish oil Waste cooking oil	Do not require land Do not affect food security Cheap feedstock Contribute to sanitation Avenues for waste to fuel Algae useful for water purification Feedstocks can be engineered	Costly production process High energy consumption Expensive oil extraction process Commercial production is not sustainable Underdeveloped technologies
Fourth	Solar biodiesel Algae	Microalgae Synthetic cell Electronbiofuel Waste cooking oil	Low carbon emission Energy security Increased carbon entrapment ability High oil contents Better cultivation, extraction, and production process	High initial investment More efforts are needed in R&D High energy requirement Research at infancy stage

**Table 3:** Examples, pros, and cons of classes of catalysts for the transesterification process.

Class of Catalyst	Examples	Pros	Cons	Ref.
Homogeneous	Base: NaOH, KOH, NaOCH_3_, KOCH_3_, NaOCH_2_CH_3_	Strong catalytic activity Fast reaction Less energy requirements Mild reaction conditions Economically viable Readily available Not corrosive	Not suitable for oil with a high FFA Possible soap formation Low biodiesel yield Requires excessive washing Requires water for purification Large volume of wastewater generated Not reusable	[33,34]
	Acid: H_2_SO_4_, HCl	Strong catalytic activity Suitable for oil with a high FFA Not affected by oil FFA and water Effective with low-grade oil Esterification and transesterification occur simultaneously No soap formation High product yield	Slow reaction rate Equipment corrosion Complex separation process Separation and reuse of the unused catalyst	[35,36]
Heterogeneous	Base: CaO, Mg/Zr, Mg-Al hydrotalcite, ZnO/KF, ZnO/Ba, Na/BaO, MgO, Al_2_O_3_/ZrO_2_/WO_3_	Reusability Easily separatable Fast reaction rate Reaction occurs in moderate conditions Low energy consumption Long catalyst life Non-corrosive Comparatively cheap Minimum effluent generation	Prone to saponification Generate more wastewater Complex separation and purification process Sensitive to the acid value of oil Low biodiesel yield Require a high methanol/oil molar ratio High cost of catalyst synthesis	[37,38]
	Acid: Titanium-doped amorphous zirconia, sulfated zirconia, carbon-basedsolid acid catalyst	Insensitive to the water content of the feedstock Effective with waste oil Easy to separate from product High reusability Highly recyclable Spent catalyst can be reused	Slow reaction rate Expensive Long residence time High energy requirements Likelihood of product contamination Leaching of catalyst Limited diffusion	[39,40]
Biobased	Lipase, candida Antarctica, immobilized lipase on SiO_2_	Completely bio-based Prevent saponification Environmentally friendly Ecofriendly and nonpolluting Easy product removal Easy purification needed Requires low temperature Zero by-product High reusability	Expensive Slow reaction rate Sensitive to methanol Can easily become inactive and denatured Complexity of separation and purification	[41,42,43]
Nanocatalyst	Zn, Ca, Mg, Zr-based nanocatalyst	Highly active Strong stability Moderate reaction conditions High reusability Strong resistance to saponification	High cost of synthesis	[44,45]

**Table 4:** Advantages and disadvantages of biodiesel production techniques [6,9,21,24,26,27].

Production Techniques	Advantages	Disadvantages
Dilution	Easy to produce Does not cause pollution Low capital and production cost	Products suffer from low volatility, poor atomization, and high viscosity Causes the plugging of injector nozzles and fuel lines Results in incomplete fuel combustion and increased pollution Increased emission of smoke and CO High engine wear and low engine durability Gum formation Oil deterioration High cost of engine maintenance Lubricating oil thickening Inappropriate for CI engine Products coagulate at low temperatures High free fatty acid
Microemulsion	Lower NOx emissions Generation of fuel with reduced viscosity and better liquidity No generation of derivatives Generation of quality fuel	Improper and incomplete combustion Deposition of carbon residue in the engine Occasional injector needle sticking Thickening of lubricating oil
Pyrolysis	Highly versatile process Easy process Satisfactory physicochemical properties of products Generation syngas and other value-added by-products High product yield	High production cost Complex equipment requirement High cost of equipment Low oxygen content of the product Involves elevated temperatures Product contains sulfur No environmental benefits High carbon residue Lower fuel purity
Transesterification	Simple process Allows feedstock flexibility Moderate production conditions Product meets international standards Lower operation cost Industrial-scale production Properties of biodiesel produced similar FB diesel fuel Flexibility in catalyst selection	Several separation processes needed High moisture content in product Generation of adulterated product Expensive catalysts Production of wastewater
Superfluid/ supercritical	Fast reaction rate High conversion efficiency No need for a catalyst Production efficiency Low cost Energy-efficient process	High cost of apparatus High reaction temperature and pressure Denatured biodiesel generated

**Table 5:** Merits and demerits of batch, semi-batch, and continuous reactors.

Reactor Modes	Process Description	Merits	Demerits	Ref.
Batch	A specified quantity of reactants is allowed into the reactor No materials added during the process The entire slurry is emptied at the end of the process	Simple to operate Monitoring of inflow and outflow of materials Can be upscaled Adequate mixing of the reactants Good flexibility Enough residence time for product formation	High operation cost High energy consumption Large space requirements Slow process Highly laborious Product quality depends on each batch Long residence time	[16,55,56,57]
Semi-batch	Intermittent addition or removal of reactants or products during the process Variation of production parameters during the reaction	High production rate Easy monitoring Better control Reduce material wastage Highly flexible Improved production rate Moderate space requirements Moderate operation cost Better heat transfer Faster production reaction High selectivity	Expensive operation cost High energy consumption Lower versatility Highly strenuous Labor-intensive process	[60,61,62,63]
Continuous	Continuous inflow of raw materials Continuous outflow of finished products Presence of mechanisms for the control of reactants addition and residence time	Low cost of operation Low space requirement Production of quality product Less energy consumption Better heat and mass transfer Fast rate of reaction	Opportunity to scale up High selectivity Low versatility High initial cost of automation technologies	[64,65]

**Table 6:** Benefits and drawbacks of tubular reactors.

Tubular Reactor Type	Benefits	Drawbacks	Ref.
Packed bed	Compatibility with an elevated temperature and pressure High conversion efficiency Better product yield Easy and simple to operate Cost effective Safety	Prone to clogging and wall erosion Difficult to monitor and control the temperature	[76,98]
Fluidized bed	Effective mixing Compatible with batch and continuous modes Low chance of tube clogging Uniform temperature Improved heat and mass transfer Easy feeding of catalysts	Expensive to build and operate Large sudden pressure drops Catalyst attrition Reactor wall erosion and corrosion	[99,100]
Trickle bed	Effective product separation Low catalyst attrition Simple to operate Ease of catalyst separation	Ineffective control of reaction parameters Difficult scalability Prone to clogging and wall erosion	[81,101]
Oscillatory flow	Low construction and running costs High product yield Compatible with batch and continuous modes Effective mixing Improved heat and mass transfer	Complex design Not mature for industrial applications	[86,90]
Micro-channel	High product yield Maximum mixing achievable Low maintenance Easy to clean and operate Improved product quality	High cost of construction Longer lengths of tubes	[102,103]

**Table 7:** Biodiesel production using tubular reactor technology.

Reactor Type	Feedstock	Catalyst Type (Dosage)	Alcohol (Dosage) ^a^	Rt (h) ^b^	RT (°C)	Yield (%)	Highlights	Ref.
Packed bed	WCO	CaO (0.5 wt.%)	Methanol (6:1)	4	65	98.40	High product yield Moderate reaction conditions	[104]
	Linseed oil	CaO (160 g)	Methanol (9.48:1)	3	30	98.08	High biodiesel yield Product of a high quality	[72]
	Coconut waste oil	Solid coconut waste (2.29 wt.%)	Methanol (12:1)	3	61	95	Product meets international standards High biodiesel yield	[105]
	WCO	Cockle shells (20 g)	Methanol (9:1)	0.75	65	72.5	Short reaction duration Moderate reaction conditions	[106]
	Palm oil	Ethyl acetate (6 wt.%)	Ethanol (16.7:1)	72	113	99	High product yield Low catalyst dosage	[107]
	Palm oil	waste seashells (10 wt.%)	Methanol (30:1)	3	65	95	High product yield Short reaction time	[108]
Fluidized bed	WCO	Magnetic whole-cell biocatalysts (12 wt.%)	Methanol (3.74:1)	48	35	91.8	High product yield Use of biocatalyst	[109]
	Soybean oil	Magnetic chitosan microspheres (25 g)	Methanol (4:1)	72	35	82	Effective use of biocatalyst Low reaction temperature	[110]
	Babassu oil	Novozym biocatalyst (12 wt.%)	Ethanol (12:1)	8	50	98.1	High biodiesel yield Simultaneous glycerol separation	[75]
	Waste frying oil	Magnetic whole-cell biocatalysts (16 wt.%)	Methanol (4:1)	48	35	89	High conversion efficiency Product ASTM D6751 and EN 14214 standards	[111]
Trickle bed	Rapeseed oil	Ca/Al oxide composite (73.8 g)	Methanol (3:1)	NS	65	94.65	Simultaneous biodiesel and glycerol separation High biodiesel yield	[112]
	Sunflower oil	CaO (18.5 g)	Methanol (2.9:1)	5	140	98	High product yield Easy separation of methanol and glycerol from biodiesel	[113]
	Palm oil	Dolomitic rock (130 g)	Methanol (12.9:1)	6	100	92.3	Improved biodiesel yield High glycerol purity (93.6 wt%) Recovery of excess methanol Easy removal of glycerol	[83]
Oscillatory flow	WCO	NaOH (1 wt.%)	Methanol (6:1)	1	60	72.5	Product of a high standard Product performed well in diesel engine Easy separation and removal of glycerol	[114]
	Palm fatty acid distillate (PEAD)	Modified sulfonated glucose (2.5 wt.%)	Methanol (9:1)	0.83	60	94.21	High product yield High conversion efficiency (97.1%) Biodiesel complied with ASTM D6751 standards	[115]
	WCO	KOH (1 wt.%)	Methanol (6:1)	5 min	60	81.9	Energy efficient process Moderate reaction conditions	[116]
	Rapeseed oil	KOH (1.5 wt.%)	Methanol (6:1)	10 min	60	97	Product meets international standard Moderate production conditions Low energy consumption Good mixing of the slurries	[117]
	WCO	KOH (3 wt.%)	Methanol (11:1)	1 min	65	99.7	High product yield Short residence time Biodiesel complied with ASTM D6751 standards	[118]
Micro-channel	Soybean oil	CaO (5 wt.%)	Methanol (12:1)	5	65	52	Less energy consumption Better mixing	[119]
	Soybean oil	NaOH (1.2 wt.%)	Methanol (9:1)	28 s	56	99.5	Improved mass and heat transfer	[120]
	Palm oil	KOH (3.5 wt.%)	Methanol (21:1)	3 min	60	100	100% product yield Short residence time Efficient mass and heat transfer	[121]
	Soybean oil	KOH (1.17 wt.%)	Methanol (8.5:1)	14.9 s	59	99.5	Short residence time High product yield	[103]
	Palm oil	KOH (1 wt.%)	Methanol (6:1)	5 s	60	97.14	High biodiesel yield Short reaction duration	[122]

^a^ Alcohol: oil ratio; ^b^ Rt = Residence time in h, unless otherwise stated; RT = Reaction temperature; WCO = Waste cooking oil; CaO = Calcium oxide; NS = Not stated.

## Abbreviations

ASTM	American Society for Testing and Materials
CI	Compression ignition
DG	Diglyceride
FAEE	Fatty Acid Ethyl Ester
FAME	Fatty Acid Methyl Ester
FB	Fossil-based
FFA	Free fatty acid
GHG	Greenhouse gas
GL	Glycerol
ICE	Internal combustion engine
MG	Monoglyceride
OECD	Organization for Economic Cooperation and Development
R & D	Research and Development
RT	Reaction temperature
Rt	Residence time
TG	Triglyceride
TWh	Terawatt hour
WCO	Waste cooking oil
